# Eating Negations

**Published:** 1872-11

**Authors:** 


### EATING NEGATIONS.

Never eat when you are not hungry.
Never eat when you are very tired.
Never eat just before severe mental or physical effort.
Never eat while in a passion.
Never eat when very low spirited.
Never eat just before a bath.
Never eat while greatly worried.

